# Hydrate of Chloral

**Published:** 1870-09

**Authors:** James C. Bascom

**Affiliations:** McLean, Ill.


ARTICLE III.—*Hydrate of Chloral.* By JAS. C. BASCOM, M.D., McLean, Ill.

For the last four months I have been using Hydrate of Chloral in my practice, and in nearly every instance with success. The indication for its use has been, with me, when Opium was indicated. The dose has been varied, according to the circumstances, the amount of pain, the character of the pain, etc. In common nervous headache, 10 grs. have almost invariably procured calm, natural sleep in ten to twenty minutes, lasting from one hour to all night—patient waking refreshed and well. In neuralgia, from 20 to 40 grs., in 10-gr. doses, every half-hour or hour, until sleep was produced. In severe cases of neuralgic rheumatism, gout, or inflammatory rheumatism, requiring from 20 to 40 grs. to procure a night's rest. In hysteria, 30 grs., to arrest the paroxysm and procure sleep. In delirium tremens, 2 drachms before sleep was induced. Acts finely in chorea, in all manner of nervous and spasmodic diseases, also whenever desirable to relieve pain of whatever character, I have used it, and generally with good results, and in no instance with any serious evils. When given in very large doses, protracted sleep was the result, and for two or three days a dull, drowsy sensation, but, with its effects, all untoward symptoms passed off. In one instance, I gave 10 grs. to a woman in the second stage of labor, a primapara, pains very frequent, os rigid, woman restless and very much fatigued; the effect was to procure twenty minutes quiet sleep, at which time the pains began to recur lightly, and in one hour were stronger than before. I thought I lost an hour, but *perhaps* saved the woman from convulsions. From my experience with Chloral, I consider it a very great acquisition to our list of remedial agents.

